# Repurposing pentamidine for cancer immunotherapy by targeting the PD1/PD-L1 immune checkpoint

**DOI:** 10.3389/fimmu.2023.1145028

**Published:** 2023-05-02

**Authors:** Tingxuan Gu, Xueli Tian, Yuanyuan Wang, Wenqian Yang, Wenwen Li, Mengqiu Song, Ran Zhao, Mengqiao Wang, Quanli Gao, Tiepeng Li, Chengjuan Zhang, Joydeb Kumar Kundu, Kangdong Liu, Zigang Dong, Mee-Hyun Lee

**Affiliations:** ^1^ Department of Pathophysiology, School of Basic Medical Sciences, Academy of Medical Science, College of Medicine, Zhengzhou University, Zhengzhou, China; ^2^ China-US (Henan) Hormel Cancer Institute, Zhengzhou, China; ^3^ Department of Immunology, The Affiliated Cancer Hospital of Zhengzhou University and Henan Cancer Hospital, Zhengzhou, China; ^4^ Department of Pathology, The Affiliated Cancer Hospital of Zhengzhou University, Zhengzhou, China; ^5^ Li Ka Shing Applied Virology Institute, University of Alberta, Edmonton, AB, Canada; ^6^ Henan Provincial Cooperative Innovation Center for Cancer Chemoprevention, Zhengzhou University, Zhengzhou, China; ^7^ College of Korean Medicine, Dongshin University, Naju, Republic of Korea

**Keywords:** PD-1/PD-L1 signaling, pentamidine, immune checkpoint, immunotherapy, cancer, anti-cancer, drug repurposing

## Abstract

Immunotherapy has emerged as an effective therapeutic approach to several cancer types. The reinvigoration of tumor-infiltrating lymphocyte-mediated immune responses *via* the blockade of immune checkpoint markers, such as program cell death-1 (PD-1) or its cognate ligand PD-L1, has been the basis for developing clinically effective anticancer therapies. We identified pentamidine, an FDA-approved antimicrobial agent, as a small-molecule antagonist of PD-L1. Pentamidine enhanced T-cell-mediated cytotoxicity against various cancer cells *in vitro* by increasing the secretion of IFN-γ, TNF-α, perforin, and granzyme B in the culture medium. Pentamidine promoted T-cell activation by blocking the PD-1/PD-L1 interaction. *In vivo* administration of pentamidine attenuated the tumor growth and prolonged the survival of tumor-bearing mice in PD-L1 humanized murine tumor cell allograft models. Histological analysis of tumor tissues showed an increased number of tumor-infiltrating lymphocytes in tissues derived from pentamidine-treated mice. In summary, our study suggests that pentamidine holds the potential to be repurposed as a novel PD-L1 antagonist that may overcome the limitations of monoclonal antibody therapy and can emerge as a small molecule cancer immunotherapy.

## Graphical Abstract

The FDA-approved anti-malarial drug pentamidine was identified as a PD-L1 inhibitor. Pentamidine can directly bind to the PD-L1 protein and induce a T-cell anti-cancer response. Furthermore, pentamidine facilitates alteration of the tumor microenvironment in the murine allograft tumor model.

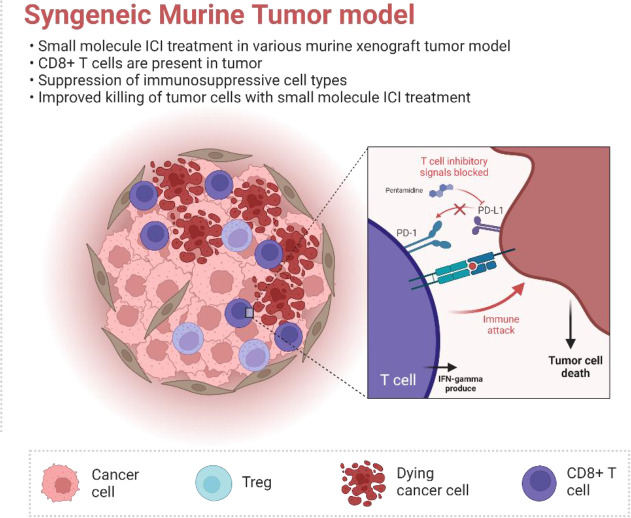

## 1 Introduction

Cancer immunotherapy has emerged as a promising frontier in anti-cancer drug discovery. The concept is based on the hypothesis that the expression of specific tumor-associated proteins, known as neoantigens, could trigger a targeted immune response against tumor tissues. This approach harnesses the body’s immune system to recognize and eliminate cancer cells, paving the way for novel therapeutic strategies (1). However, one of the key hallmarks of cancer is immune evasion, whereby the tumor escapes the host’s immune response and continues to grow unabated (2). Moreover, the question of how cancer escapes immune surveillance and continues to grow has been actively investigated over recent years (3, 4). Tumor cells often attack T cells, causing T-cell destruction and dampening the body’s immune response to the growing tumor. Mechanistically, T cells are equipped with several cell surface inhibitory receptors, such as cytotoxic T lymphocyte-associated protein-4 (CTLA4) and program cell death receptor-1 (PD-1), which signal T-cell apoptosis as a means to avoid autoimmunity (5). It was shown that the interaction between CTLA4 and B7.1 inhibits T-cell proliferation while the binding of PD-1 with its cognate ligand PD-L1 promotes T-cell apoptosis. Over time, tumor cells develop mechanisms to activate T-cell inhibitory signals, causing T-cell exhaustion and leading to immune escape (6). Thus, one of the strategies in designing cancer immunotherapies is to block the activation of CTLA4 and PD-1. This invigorates anergic T cells able to launch an immune response against tumor tissue (7). In line with this thought, the approval of ipilimumab, a monoclonal antibody against CTLA4, for the treatment of late-stage melanoma in 2011 by the US Food and Drug Administration (FDA) has been the breakthrough in the emergence of cancer immunotherapy (8). Subsequently, several monoclonal antibodies (e.g., nivolumab and pembrolizumab) against PD-1 and others (atezolizumab and durvalumab) against PD-L1 received FDA approval as immunotherapies for various cancer types, including melanoma, renal cell carcinoma, non-small cell lung cancer, head and neck cancer, and stomach cancer (9).

Despite this remarkable progress in cancer immunotherapies, multiple lines of evidence indicate that antibody therapies targeting immune checkpoint markers, such as CTLA4, PD-1, or PD-L1, often lead to severe adverse drug reactions resulting from an elevated immune response (10). Moreover, the production, distribution, storage, and route of administration of antibody-based immunotherapy require special considerations. Therefore, designing small molecule inhibitors to target CTLA4, PD-1, or PD-L1 would be a rational approach to blocking inhibitory immune signaling that contributes to T-cell exhaustion (11). Over the last decades, extensive immuno-oncology research has revealed that tumor cell-derived PD-L1 interacts with PD-1 located on the T-cell surface, resulting in T-cell anergy. Thus, a blockade of the interaction between PD-1 and PD-L1 would maintain T-cell activation and facilitate an anti-tumor immune response (12). With the elucidation of the crystal structure of PD-L1, it is now possible to design small molecules able to block the interaction between PD-L1 and PD-1 (13). In the present study, we conducted a virtual docking analysis of small molecules and identified pentamidine as a potential candidate small molecule that binds to PD-L1. Pentamidine is a well-known antimicrobial agent commonly used in treating African trypanosomiasis, leishmaniasis, and pneumocystis pneumonia in people with impaired immune function (14, 15). Pentamidine also inhibits prostate and ovarian cancer cell proliferation (16, 17). Our study revealed that pentamidine restores T-cell activity, enhances T-cell-mediated anti-cancer cytotoxicity, and inhibits *in vivo* tumor growth by interfering with the interaction between PD-1 and PD-L1.

## 2 Materials and methods

### 2.1 Cell culture

H1975, A375, H1937, HCT116, Jurkat, and HEK 293T cells were purchased from Cell Bank Australia (Shanghai, China). Murine cancer cell lines 4T1, KLN205, MC38, and B16F10 were purchased from Cobioer Bio (Nanjing, China). H1975 and Jurkat cells were maintained in RPMI-1640 (Cat#01-100-1ACS, Biological Industries, Kibbutz Beit-Haemek, Israel) supplemented with 10% FBS (Cat#04-001-1ACS, Biological Industries, Kibbutz Beit-Haemek, Israel) and 100 U/ml penicillin/streptomycin (Cat#P1400, Solarbio, Beijing, China). A375, HEK293T, 4T1, MC38, and B16F10 cells were maintained in DMEM (Cat#01-052-1ACS, Biological Industries, Kibbutz Beit-Haemek, Israel) supplemented with 10% FBS and 100 U/ml penicillin/streptomycin. HCT116 cells were maintained in McCoy’s 5A medium (Cat#01-075-1ACS, Biological Industries, Kibbutz Beit-Haemek, Israel) supplemented with 10% FBS and 100 U/ml penicillin/streptomycin. H1937 cells were maintained in RPMI-1640 supplemented with 10% FBS, 100 U/ml penicillin/streptomycin, 0.1 mM non-essential amino acids (Cat#11140076, Gibco, Grand Island, New York), and 1 mM sodium pyruvate (Cat# 11360088, Gibco, Paisley, Scotland, UK). KLN205 cells were maintained in MEM (Cat#01-040-1ACS, Biological Industries, Kibbutz Beit-Haemek, Israel) supplemented with 10% FBS, 100 U/ml penicillin/streptomycin, 0.1 mM non-essential amino acids, and 1 mM sodium pyruvate. All cell lines were authenticated by STR profiling provided by Genewiz (GENEWIZ, Inc., Suzhou, China).

### 2.2 Isolation and culture of human PBMCs and culture

Human PBMCs were purchased from TPCS (Milestone Biotechnologies, Shanghai, China). CD3+ T cells were isolated from PBMCs by CD3 magnetic negative selection using the EasySep Human T Cell Isolation Kit (Cat#17951, STEMCELL Technologies, Cologne, Germany) according to the manufacturer’s instructions. T cells were stimulated as described below or after thawing from cell stock. When frozen T cells were used, previously isolated PBMCs had been frozen in 90% FBS and 10% DMSO and cultured in X-VIVO™ 15 serum-free hematopoietic cell medium (Biowhittaker, Walksville, MD) supplemented with 200 U/ml IL-2 (Cat# PHC0026, Gibco, Grand Island, New York). After thawing, the cells were cultured in medium supplemented with 5% FBS and maintained without stimulation for 1 day. The cells were then stimulated as described below. Unless otherwise stated, human primary T cells were cultured in XVivo-15 medium supplemented with 5% FBS and 200 U/ml IL-2. T cells were stimulated for 48 h by anti-CD3/CD28 magnetic dynabeads (Cat# 11131D, Gibco, Grand Island, New York) at a ratio of 1:1 together with 200 U/ml IL-2, 50 ng/mL IL-7 (Cat# PHC0075, Gibco, Grand Island, New York), and 50 ng/ml IL-15 (Cat# PHC9151, Gibco, Grand Island, New York). After stimulation, CD3+ T cells were maintained in the previously described culture medium at a density of 1 million cells per ml of culture medium. The culture medium was replaced every 2–3 days.

### 2.3 Plasmid construction

TCR activator and TCR activator + PD-L1 plasmids were purchased from BPS Bioscience, Inc. Human PD-1 and PD-L1 ORFs were purchased from Sino Biological Inc. The pLenti-IRES-EGFP-PD-1 plasmid was established from pLenti-IRES-EGFP using the Xba1 and BamH1 restriction sites. The pLenti-NFAT-IRES-EGFP-PD-1 plasmid was established from pLenti-IRES-EGFP-PD-1 and pGL3-NFAT luciferase (addgene# 17870) using the EcoR1 restriction site to linearize pLenti-IRES-EGFP-PD-1; the following primers were used for cloning:

ASM-NFAT-F: cgattagtgaacggatcttccatcgTGTATCCCCACCCCCCCTCG
ASM-NFAT-R: gatgaatactgccatttgtctgcagTTACACGGCGATCTTTCCGCCC

The 3*NFAT-mini-IL2 promoter-Luciferase expression cassette was amplified, and the expression cassette was inserted into the linearized pLenti-IRES-EGFP-PD-1 using the Gibson Assembly method.

The pLenti-NFAT-IRES-EGFP plasmid was generated from the pLenti-NFAT-IRES-EGFP-PD-1 construct by removing the human PD-L1 ORF. This was achieved through digestion with Xba1 and BamH1 restriction enzymes at specific sites. Following digestion, the Taq enzyme was used to fill in the overhangs and create blunt ends, and the T4 ligase was employed to repair the junction, producing the pLenti-NFAT-IRES-EGFP plasmid.

The pCDH-CMV-MCS-EF1α-copGFP-T2A-Puro-PD-L1 (human) (pCDH-hPD-L1) plasmid was generated from the pCDH-CMV-MCS-EF1α-copGFP-T2A-Puro construct by inserting the human PD-L1 ORF. This was accomplished using Xba1 and EcoR1 restriction enzymes to create compatible ends at the specific sites within the vector, allowing for the successful insertion of the human PD-L1 ORF.

The pLenti-IRES-EGFP-PD-L1 plasmid was generated by first linearizing the pLenti-IRES-EGFP plasmid at the Xba1 and BamH1 restriction sites. The human PD-L1 ORF was amplified using the following primers:

ASM-PD-L1-F: cgcttcgaaggtacctATGAGGATATTTGCTGTCTTTATATTCATGACCT
ASM-PD-L1-R: ggagggagaggggcgTTACGTCTCCTCCAAATGTGTATCACTTTGC

Subsequently, the amplified human PD-L1 ORF was inserted into the linearized pLenti-IRES-EGFP vector using the Gibson Assembly method, resulting in the creation of the pLenti-IRES-EGFP-PD-L1 plasmid.

### 2.4 CRISPR/Cas9 sgRNA construction

CRISPR/Cas9 single guide RNA (sgRNA) was designed using the CHOPCHOP website (http://chopchop.cbu.uib.no/; Labun, Kornel, et al.). Three sgRNA were designed against mouse PD-L1 gene locus exons 1 and 2. Oligo DNA to generate sgRNA was synthesized by GENEWIZ (Suzhou, China). The sgRNA oligo was annealed and cloned into the BsmB1 site of the lentiCRISPR v2 vector using T4 ligase. The ligation product was transformed into stbl3 competent cells. SgRNA inserts were verified by Sanger sequencing.

To generate the lentivirus, Lenti-X-293T cells (Takara Biomedical Technology Co., Ltd., Beijing, China) were grown to approximately 70% confluence prior to transfection. The LentiCRISPRv2 plasmid for murine PD-L1 knockout, the human PD-L1 expression plasmid PCDH-CMV-MCS-copEGFP-T2A-PURO, and pLenti-IRES-EGFP were transfected using X-fect transfection reagent (Cat#631318, Takara Biomedical Technology Co., Ltd., Beijing, China) according to the manufacturer’s protocol. Six hours after transfection, the medium was replaced with fresh complete growth medium. The virus-particle-enriched medium was subsequently harvested after 48 h of incubation. The medium was filtered through a 0.45-µm filter and centrifuged in an ultracentrifuge at 20,000 rpm for 2 h prior to infecting the target cells.

### 2.5 *In vitro* affinity assays

The affinity of pentamidine with PD-1 or PD-L1 proteins was measured using surface plasmon resonance (SPR) experiments performed with a BIACORE T-200 (GE Healthcare, UK) equipped with a research-grade CM5 sensor chip (GE Healthcare, UK). PD-1 (Cat#10377-H08H, Sino Biological, Beijing, China) or PD-L1 (Cat#10084-H08H, Sino Biological, Beijing, China) proteins were immobilized using an amine-coupling kit. In brief, the flow cell surfaces were activated with a mixture of NHS (N-hydroxysuccinimide) and EDC (1-ethyl-3-(3-dimethylaminopropyl)-carbodiimide hydrochloride). Next, 20 ng/μl of protein diluted in 10 mM sodium acetate (pH 4.5) was immobilized in the activated channel. After immobilization, the channel was blocked with 1 M ethanolamine (pH 8.0). To calculate affinity, pentamidine suspended in PBS (pH 7.4) was injected at several concentrations at a flow rate of 30 μl/min at 25°C. Pentamidine was allowed to contact and dissociate for 120 and 600 s, respectively. Approximately 15 μl of glycine buffer (10 mM, pH 2.5) was then used to regenerate the surface. For each sample sensorgram, the relative response was collected, and the blank was subtracted.

### 2.6 T-cell cytotoxicity assay

Primary T cells were cultured and expanded as described below. Primary T-cell cytotoxicity to cancer cells was examined using a CFSE/PI labeled cytotoxicity assay. Cancer cells were labeled with CFSE (Invitrogen) according to the manufacturer’s instructions. CFSE-labeled cancer cells were seeded into a 24-well plate and allowed to adhere overnight. The labeled cells were then incubated with primary T cells at a T cell to cancer cell ratio of 5:1 for 18 h. PI staining was used to determine dead cancer cells *via* flow cytometry analysis.

### 2.7 *In vitro* cytokines production assay

The production of cytotoxicity-related cytokines in the supernatant was measured using an ELISA assay according to the manufacturer’s instructions. The secretion levels of tumor necrosis factor-α (TNF-α) (Cat# KIT10602, Sino Biological, Beijing, China), interferon gamma (IFN-γ) (Cat# KIT11725A, Sino Biological, Beijing, China), Granzyme B (Cat# 1118502, Dakewe Bio-engineering Co., Ltd., Shenzhen, China), and perforin (Cat# 1118302, Dakewe Bio-engineering Co., Ltd., Shenzhen, China) was measured using a cytokine ELISA kit.

### 2.8 PD-L1 humanization of mouse cell line

For the generation of the lentiviral-based PD-L1 humanized murine cell line (4T1, MC38, KLN205, and B16F10), we first established PD-L1 knockout cells *via* the CRISPR/Cas9 system to disrupt the murine PD-L1 ORF. sgRNAs specifically targeting and generating frameshift murine PD-L1 gene loci were established using the CHOPCHOP web-based software (https://chopchop.rc.fas.harvard.edu/). An all-in-one lentiviral vector (LentiCRISPRv2, addgene#52961) was chosen to generate the Cas9 and sgRNA expression vector for murine PD-L1 knock-out. The sgRNAs for murine PD-L1 are as follows:

Sg-1: CATAATCAGCTACGGTGGTG
Sg-2: TGAACTAATATGTCAGGCCG
Sg-3: GACTTGTACGTGGTGGAGTA
Sg-4: CTCTCCAGATACACAATTCG

The oligomers used are as follows:

mCD274-1F CACC GCATAATCAGCTACGGTGGTG
mCD274-1R AAAC CACCACCGTAGCTGATTATGC
mCD274-2F CACC GTGAACTAATATGTCAGGCCG
mCD274-2R AAAC CGGCCTGACATATTAGTTCAC
mCD274-3F CACC GACTTGTACGTGGTGGAGTA
mCD274-3R AAAC TACTCCACCACGTACAAGTC
mCD274-4F CACC GCTCTCCAGATACACAATTCG
mCD274-4R AAAC CGAATTGTGTATCTGGAGAGC
sgRNAs were subcloned into the LentiCRISPRv2 plasmid using the BsmB1 site. After generation, the following primers were used for Sanger sequencing: LKO.1 5’: GACTATCATATGCTTACCGT.

The sequenced plasmids were used to generate lentiviruses as described above. After infection for 48 h, murine cells were selected by puromycin (2 µg/ml final) and subsequently analyzed by flow cytometry.

Human PD-L1 was subcloned into the PCDH-CMV-MCS-copEGFP-T2A-PURO and pLenti-IRES-EGFP vectors as described below. The lentivirus carries human PD-L1 ORF and infects murine PD-L1 knock-out cells; after 48 h, the PD-L1 humanized cells were verified with flow cytometry as described below.

### 2.9 Mice model

To establish syngeneic allograft models, male and female C57BL/6, BALB/c, and DBA/2 mice (18–20 g, aged 5–6 weeks) were first purchased from Beijing Vital River Laboratory Animal Technology Co., Ltd. (Beijing, China). Approximately 2 million 4T1 PD-L1 humanized cells were subcutaneously injected (s.c.) into female Balb/c mice; 2 million MC38 or B16-F10 PD-L1 humanized cells were subcutaneously injected into male C57/BL6 mice; and 5 million KLN205 PD-L1 humanized cells together with 1:1 matrigel matrix (Cat#354234, Corning, NY, USA) were subcutaneously injected into female DBA/2 mice. After the average tumor volume reached roughly 100 mm^3^, the animals were treated with pentamidine or atezolizumab by i.p. administration until the average tumor volume reached approximately 1,000 mm^3^. All animal experiments were performed following institutional guidelines and approved by the China-US (Henan) Hormel Cancer Institute Ethics Committee (following internationally established guidelines, CUHCI2017007).

### 2.10 Multiplex cytokines assay

Mice blood was collected from the abdominal aorta after euthanization. Blood serum was separated by centrifugation and stored at −80 °C until needed. Next, concentrations of IL-1α, IL-1β, IL-6, IL-10, IL-12p70, IL-17A, IL-23, IL-27, MCP-1, IFN-β, IFN-γ, TNF-α, and GM-CSF were measured using the LEGENDplex™ Mouse Inflammation Panel (13-plex, Biolegend, cat#740446); data analysis was conducted using a FACSCalibur (BD) flow cytometer.

### 2.11 Tumor tissue dissociation

Tumor tissues were dissociated with 5 µg/ml collagenase II (in DMEM, Sigma-Aldrich, C685) and 60 U/ml DNase (in PBS, Sigma-Aldrich, DN25). Fragments were mechanically dissociated by spring dissection scissors on ice to generate roughly 1 mm^3^ pieces, agitated gently at 37 °C for 45 min. Cells were then filtered through a 70 μm cell strainer (Corning, Inc.); TILs were subsequently analyzed using flow cytometry.

### 2.12 Flow cytometry and cell sorting

To separate the cells, we utilized the FACSCalibur (BD), followed by the FACSAria (BD). Surface staining assays for flow cytometry and cell sorting assays were conducted. Single cells were suspended in 200 µl of PBS (containing 1% serum) with or without labeled antibodies, following the manufacturer’s protocol. The APC-labeled anti-mouse PD-L1 antibody (Cat#124312; Biolegend) and PE-labeled anti-human PD-L1 antibody (Cat#329706; Biolegend) were employed to stain human and murine PD-L1 in flow cytometry sorting. Isotype PE mouse IgG2b (Cat# 400313, Biolegend) and APC rat IgG2b (Cat# 400612, Biolegend) were used as isotype control antibodies.

### 2.13 Competitive ELISA assay

To determine the effect of test inhibitors on the PD-1/PD-L1 protein–protein interaction, PD1/PD-L1 Competitive ELISA Screening Kits (BPS Bioscience Inc., San Diego, CA, USA) were utilized per the manufacturer’s instructions. In brief, 96-well plates were coated overnight with 1 g/ml of recombinant hPD-L1 in PBS. Plates were washed with PBS containing 0.1% Tween and blocked at room temperature for 1 h with PBS containing 2% BSA. The wells were filled with 50 μl of 0.5 g/m biotinylated hPD-1 and then incubated for 2 h at RT. After three washes, 0.2 µg/ml HRP-conjugated streptavidin was added to each well, and the plates were incubated for 1 h. After incubation, the plates were washed three times. Relative chemiluminescence was subsequently evaluated using a Molecular Devices SpectraMax L Luminometer (San Jose, CA, USA).

### 2.14 Sepharose 4B bead-mediated pull down assay

In this assay, pentamidine-coupled Sepharose 4B beads were prepared by first dissolving pentamidine in a coupling buffer containing 100 mM sodium bicarbonate (pH 8.3) and 0.5 M sodium chloride; DMSO-coupled Sepharose 4B beads were performed as a control group. Sepharose 4B beads were then washed with 1 mM hydrochloric acid before being combined with the pentamidine-containing coupling buffer. This mixture was gently rotated at 4°C overnight. Unbound pentamidine was removed by washing with coupling buffer, and any remaining reactive sites on the beads were blocked with 0.1 M Tris–HCl buffer (pH 8.0) for 2 h. Subsequently, the beads were washed sequentially with 0.1 M acetate buffer (pH 4.0) and 0.1 M Tris–HCl buffer (pH 8.0), both containing 0.5 M sodium chloride. The prepared pentamidine-Sepharose 4B beads were then ready for use in pull-down assays.

For the pull-down assay, PD-1 and PD-L1 recombinant proteins (20 µg each) or H1975 cell lysate (600 µg) were incubated with either pentamidine-coupled Sepharose 4B beads or Sepharose 4B beads alone (as a negative control) in a reaction buffer composed of 50 mM Tris–HCl (pH 7.5), 5 mM EDTA, 150 mM sodium chloride, 1 mM dithiothreitol, 0.01% NP-40, 2 µg/ml bovine serum albumin, and a 1× protease inhibitor cocktail. The mixture was incubated under gentle rocking at 4°C overnight. Following incubation, the beads were washed five times using a wash buffer containing 50 mM Tris–HCl (pH 7.5), 5 mM EDTA, 150 mM sodium chloride, 1 mM dithiothreitol, 0.01% NP-40, and 0.02 mM phenylmethylsulfonyl fluoride. Finally, the proteins bound to the beads were analyzed by immunoblotting using antibodies against PD-1 or PD-L1.

### 2.15 Statistical analysis

GraphPad Prism 7 (GraphPad Software Inc., La Jolla, CA, USA) was used for performing all statistical analyses. Kruskal–Wallis, unpaired *t*-test, and one-way ANOVA statistical tests were performed in GraphPad Prism. The Bonferroni *post-hoc* test was used in multiple comparisons: as *P <0.05, **P <0.01, ***P <0.001, and ****P <0.001. Survival analysis was performed by the two-sided log-rank test.

### 2.16 Graphical illustrations

Graphical illustrations were created with (BioRender.com).

### 2.17 Study approval

Ethical approval of animal studies was received from the China-US (Henan) Hormel Cancer Institute Ethics Committee, CUHCI2017007.

## 3 Results

### 3.1 Pentamidine enhances T-cell-mediated cancer cell cytotoxicity

We first conducted a virtual screening of chemicals from a compound library using the Schrödinger Suite 2018 software to identify small molecule antagonists of human PD-1 or PD-L1. Based on the results of the *in-silico* docking experiments, we procured selected small molecule compounds to verify their respective antagonistic activities using T-cell cytotoxicity assays. Compounds that significantly increased T-cell cytotoxicity against cancer cells were selected. Out of 21 compounds screened for the T-cell cytotoxicity assay, we selected pentamidine as a candidate small molecule for further investigation. We next performed flow cytometry to assess PD-L1 expression levels in various human cancer cells to identify suitable cancer cells for a subsequent study (**Figure S1A**). We found that pentamidine did not exhibit cytotoxic effects in primary T cells or human lung cancer cells (H1975) when incubated separately at concentrations below 10 µM (**Figures S1B, C**). However, treatment of co-cultured primary human T cells and H1975 cells with pentamidine significantly enhanced the cytotoxicity of primary T cells against H1975 cells at a dose of 0.5 µM (**Figure S1C**).

**Figure 1:**
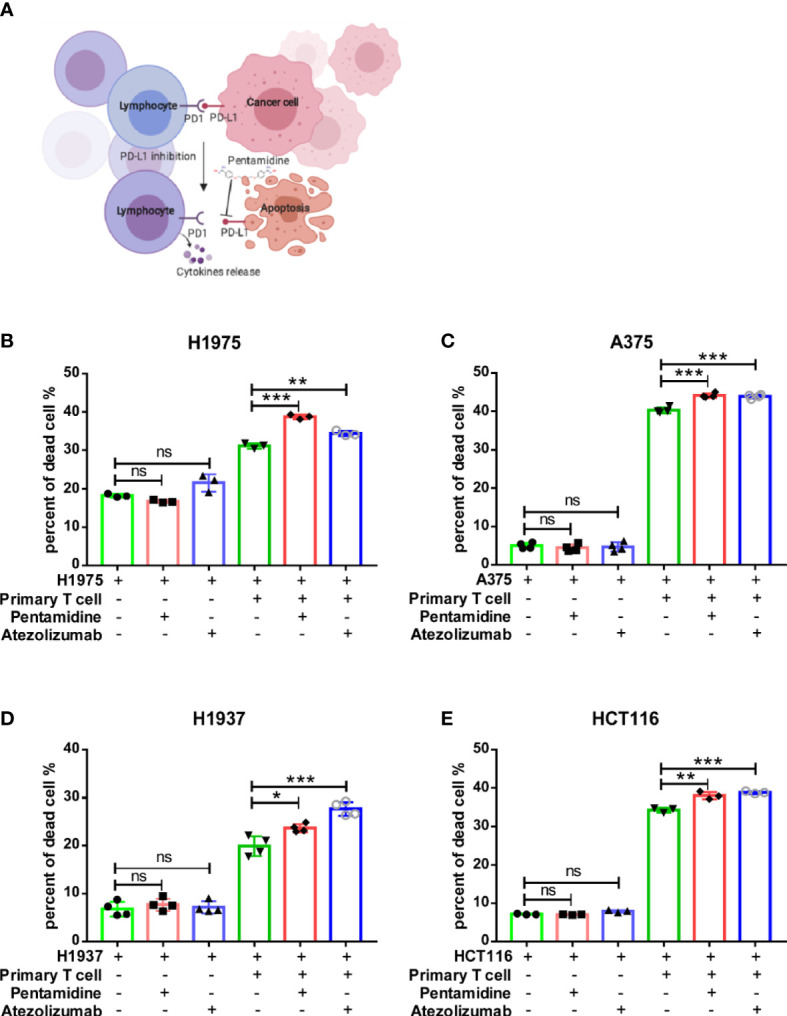
Pentamidine enhances primary T-cell anti-tumor cytotoxicity. **(A)** A schematic representation of T-cell cytotoxicity against cancer cells and cytokine release. **(B–E)** Quantification of purified primary T-cell cytotoxicity against H1975, A375, H1937, and HCT116 cancer cells upon treatment with either pentamidine (0.5 µM) or atezolizumab (1 µg/ml). *p <0.05, **p <0.01, ***p <0.001; ns, not significant; one-way ANOVA with a *post-hoc* Bonferroni test.

**Figure 2:**
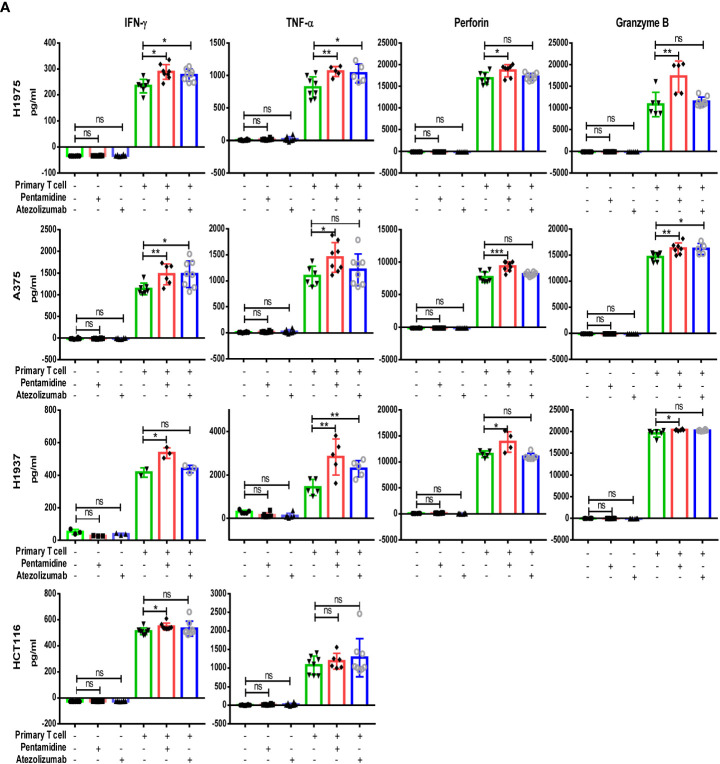
Quantification of cytokine IFN-γ, TNF-α, perforin, and Granzyme B production in co-culture medium determined by ELISA assay, **(A)** following treatment with either pentamidine (0.5 µM) or atezolizumab (1 µg/ml). *p <0.05, **p <0.01, ***p <0.001; ns, not significant; one-way ANOVA with *post-hoc* Bonferroni test.

After screening the small molecule candidates, we conducted flow cytometry assays to examine the cytotoxic effects induced by T cells. In brief, we purified CD3+ T cells from human peripheral blood mononuclear cells (PBMCs). Next, the CD3+ T cells were co-cultured with CFSE-labeled target tumor cells (H1975, A375, H1937, and HCT116) that express PD-L1. After PI staining, dead CFSE-labeled cancer cells were quantified by flow cytometry (**Figure 1A**). Incubation of co-cultured primary T cells and various cancer cells with pentamidine (0.5 µM) or atezolizumab (1 μg/ml) showed enhanced tumor cell death as compared to that induced by pentamidine or atezolizumab in the absence of T cells in the culture (**Figure 1B**). Similarly, T cell-mediated tumor cell cytotoxicity was induced by pentamidine treatment in three additional cancer cell lines (A375, H1937, and HCT116; **Figures 1C–E**) upon co-culture with T cells. We then measured several T-cell activation marker cytokines using the same co-culture system after treatment with pentamidine or atezolizumab. Results showed that the secretion of IFN-γ, TNF-α, perforin, and granzyme B in the culture medium was significantly increased in co-cultured cells treated with pentamidine or atezolizumab as compared to those treated in the absence of T cells (**Figure 2A**).

### 3.2 Pentamidine restores T-cell activity by blocking PD-1/PD-L1 interaction

To further investigate the PD-1/PD-L1 interaction, we generated a cellular reporter system to monitor T-cell activation (18, 19). A nuclear factor for activated T-cell (NFAT)-derived luciferase reporter and human PD-1 ORF were delivered by lentivirus into Jurkat cells, which we referred to as Jurakt-NFAT-PD-1 (**Figure S2A**). Transduced Jurkat cells were sorted by flow cytometry, and single clones were seeded in 96-well plates by serial dilution. Single-cell clones were stained with human PD-1 antibody and verified by flow cytometry according to the manufacturer’s protocol. Treatment of single clones with PMA (50 ng/ml), an activator of NFAT, showed that Jurakt-NFAT-PD-1 clone 3 expressed elevated PD-1 protein levels and generated a high luciferase signal (**Figures S2B, C**); therefore, Jurkat-NFAT-PD-1 clone 3 was selected for subsequent study. We then cloned an NFAT-derived luciferase reporter by removing human PD-1 from the NFAT-PD-1 expression plasmid and generated a Jurkat-NFAT cell line. At the same time, we generated the 293T-TCR activator PD-L1 cell line. In brief, TCR-activator plus human PD-L1 ORF and TCR-activator cassettes were transfected into HEK-293T cells. After Hygromycin B (500 μg/ml) selection, single-cell clones were generated. We next used flow cytometry to measure PD-L1 levels in the 293T-TCR activator-PD-L1 cell clones and identified that clone 37 expressed the highest PD-L1 protein; clone 37 was subsequently expanded and used in future experiments (**Figure S2D**). Similarly, 293T-TCR activator cells were generated as a control.

**Figure 3:**
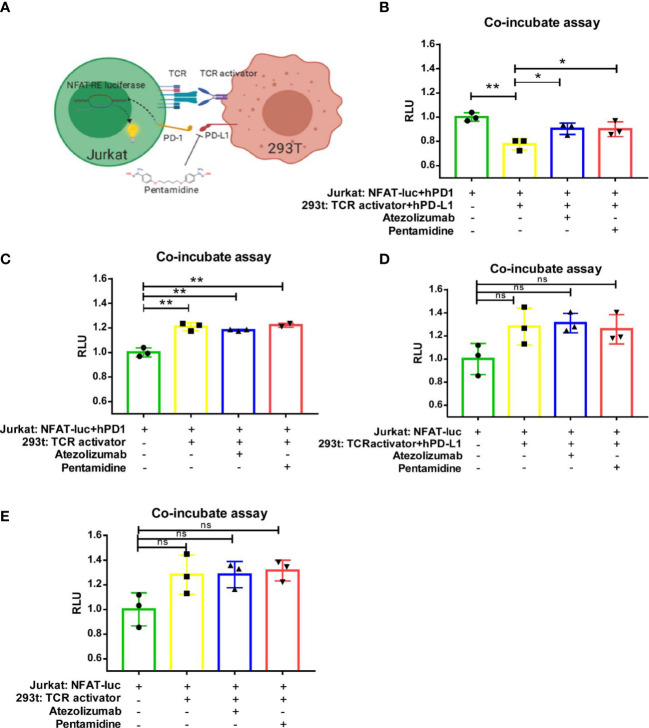
Pentamidine reverses PD-1/PD-L1-mediated TCR signaling inhibition. **(A)** A schematic illustration of the NFAT-luciferase reporter in the context of cell interactions, depicting the mechanism by which pentamidine blocks the PD-1/PD-L1 interaction to counteract PD-L1-mediated T-cell inhibition. TCR, T-cell receptor; NFAT-RE luciferase, 3×NFAT binding site-minimal IL2 promoter-firefly luciferase. **(B)** Quantification of Jurkat: NFAT-luc-PD-1 cells co-cultured with 293T: TCR activator-PD-L1, treated with either pentamidine or atezolizumab. **(C)** Quantification of Jurkat: NFAT-luc-PD-1 cells co-cultured with 293T: TCR activator, treated with either pentamidine or atezolizumab. **(D)** Quantification of Jurkat: NFAT-luc cells co-cultured with 293T: TCR activator-PD-L1, treated with either pentamidine or atezolizumab. **(E)** Quantification of Jurkat: NFAT-luc cells co-cultured with 293T: TCR activator, treated with either pentamidine or atezolizumab. *P <0.05; **P <0.01; ns, not significant; one-way ANOVA with a *post-hoc* Bonferroni test.

Theoretically, the TCR-activator expressed in HEK293T cells, which also express PD-L1, would facilitate activation of the TCR in Jurkat cells; this interaction would ultimately result in increased NFAT-derived luciferase activity in the Jurkat-NFAT-PD-1 cell line. In contrast, the interaction of PD-1 (from Jurkat NFAT-PD-1 cells) with PD-L1 (from 293T-TCR activator PD-L1 cells) could decrease the activation of TCR signaling, thereby decreasing the NFAT-derived luciferase signal. A schematic illustrating how the reporter system operates is shown in **Figure 3A**. We then co-cultured 293T-TCR activator-PD-L1 cell clone 37 (TP37) and Jurakt-NFAT-PD-1 clone 3 (NP3) in 96-well plates and treated the cells with 0.5 µM pentamidine or 1 µg/ml atezolizumab (a monoclonal antibody against PD-L1) for 6 h. As shown in **Figure 3B**, the decrease in NFAT-luc signal upon co-culture of TP37 and NP3 cells resulted from the interaction between NP3 cell-derived PD-1 and TP37 cell-derived PD-L1. However, treatment of these co-cultured cells with atezolizumab or pentamidine restored the NFAT-luc signals, suggesting that the blockade of PD-L1 restricted the PD-1/PD-L1 interaction and fostered the TCR activator–TCR interaction. We also generated reporter systems that lack PD-L1 in TP37 cells (293-TCR-activator) or PD-1 in NP3 cells (Jurkat-NFAT-Luc). While the co-culture of Jurkat-NFAT-luc-PD-1 with 293T TCR-activator (**Figure 3C**), that of Jurkat-NFAT-Luc cells with TP37 (**Figure 3D**), or that of Jurkat-NFAT-Luc cells with a 293T-TCR activator (**Figure 3E**) showed increased NFAT-luc response resulting from the engagement of TCR-activator with TCR, the incubation with pentamidine or atezolizumab failed to alter the NFAT-luc activity. These findings suggest that blocking the PD-1/PD-L1 interaction with pentamidine restores T-cell activation.

### 3.3 Pentamidine binds to human PD-L1 *in vitro*

**Figure 4:**
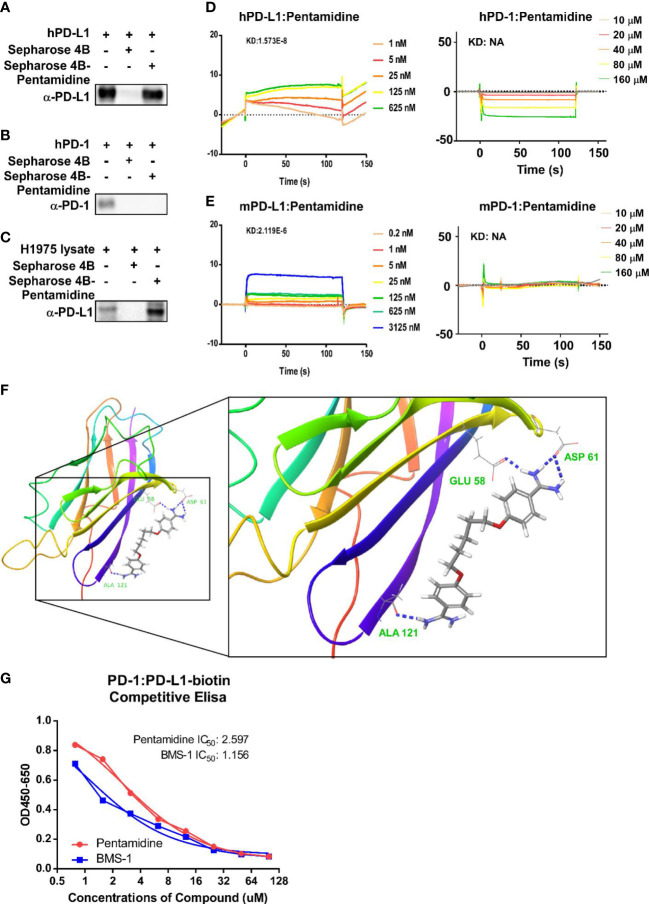
Prediction and validation of pentamidine’s binding affinity to human and mouse PD-1/PD-L1. **(A–C)** Detection of Sepharose 4B beads-conjugated pentamidine binding to recombinant human PD-L1 **(A)**, PD-1 **(B)**, and H1975 cell lysate **(C)** using Sepharose 4B beads-mediated pull-down and Western blot analysis with antibodies against human PD-L1. **(D)** Surface plasmon resonance sensorgrams examining the binding affinity of pentamidine to human PD-1 (right panel) and PD-L1 (left panel). **(E)** Surface plasmon resonance sensorgrams evaluating the binding affinity of pentamidine to mouse PD-1 (right panel) and PD-L1 (left panel). **(F)** A computational docking model of pentamidine for human PD-L1, with putative binding sites of PD-L1 to pentamidine being Asp61, Glu58, and Ala121. **(G)** A competitive ELISA assay was employed for analyzing the antagonist activity of pentamidine and BMS-1.

To verify whether pentamidine can bind with PD-1 or PD-L1, we performed a binding assay in which pentamidine was first conjugated with Sepharose 4B beads and then incubated with recombinant human-PD-1, recombinant PD-L1, or cell lysates derived from H1975 cells. The results indicated that pentamidine interacted with recombinant human PD-L1 and H1975 cell lysates but not with recombinant PD-1 (**Figures 4A–C**). The ability of pentamidine to bind to PD-1 and PD-L1 was further analyzed by SPR. Pentamidine was perfused through the sensor chip previously amine-coupled with human PD-1 or PD-L1 (**Figure 4D**) or mouse PD-1 or PD-L1 (**Figure 4E**). Pentamidine showed a concentration-dependent increase in the SPR signal (response units) when allowed to flow over human or mouse PD-L1 but failed to induce an SPR signal when passed over mouse or human PD-1 (**Figures 4D, E**). The binding kinetics analysis showed that pentamidine binds to human PD-L1 with a KD value of 1.573E−8, and 2.119E−6 with murine PD-L1. The software-based docking study revealed that pentamidine’s putative binding site is located at the Glu-58, Asp-61, and Ala-121 sites of PD-L1 (**Figure 4F**). To demonstrate the antagonistic activity of pentamidine, we utilized a competitive ELISA test with biotin-labeled PD-L1 and PD-1. The IC50 of pentamidine in the competitive assay was measured at 2.597 µg/ml, whereas the IC50 of the PD-1 inhibitor BMS-1 was measured at 1.156 µg/ml (**Figure 4G**). Taken together, these findings indicate that pentamidine exhibits affinity toward human PD-L1 and antagonistic activity in disrupting the interaction between PD-1/PD-L1 *in vitro*.

### 3.4 Pentamidine inhibits the growth of PD-L1 humanized murine cancer cells *in vivo*

The structure of the mPD-1/hPD-L1 complex was previously established (20). Moreover, murine PD-1 can bind PD-L1 of either species with similar affinities *in vitro*. There are different druggability profiles between murine PD-L1 and human PD-L1; thus, a PD-L1 humanized syngeneic tumor model is necessary for the evaluation of a human PD-L1 inhibitor (21, 22). Although the syngeneic mouse model is typically used to evaluate the effect of candidate immune checkpoint inhibitors on murine immune system markers, it limits the translation of these animal model-derived results into clinical applications. Therefore, we developed a PD-L1 humanized syngeneic mouse tumor model to evaluate the anti-cancer effect of pentamidine or atezolizumab. A schematic detailing the experimental design is illustrated in **Figure S3A**. Briefly, murine PD-L1 expressed in the murine cancer cell line was replaced by full-length human PD-L1. We then quantified murine PD-L1 and human PD-L1 expression by flow cytometry (**Figures S3B, C**). Results showed that sgRNA4 possessed the highest targeting efficiency (**Figure S3B**). Next, we applied sgRNA4 to the following four murine cell lines: murine breast cancer cell line 4T1, murine colon cancer cell line MC38, NSCLC cell line KLN205, and murine melanoma cell line B16F10. Our findings indicated that murine PD-L1 expression was nearly absent and human PD-L1 was highly expressed (**Figure S3C**).

**Figure 5:**
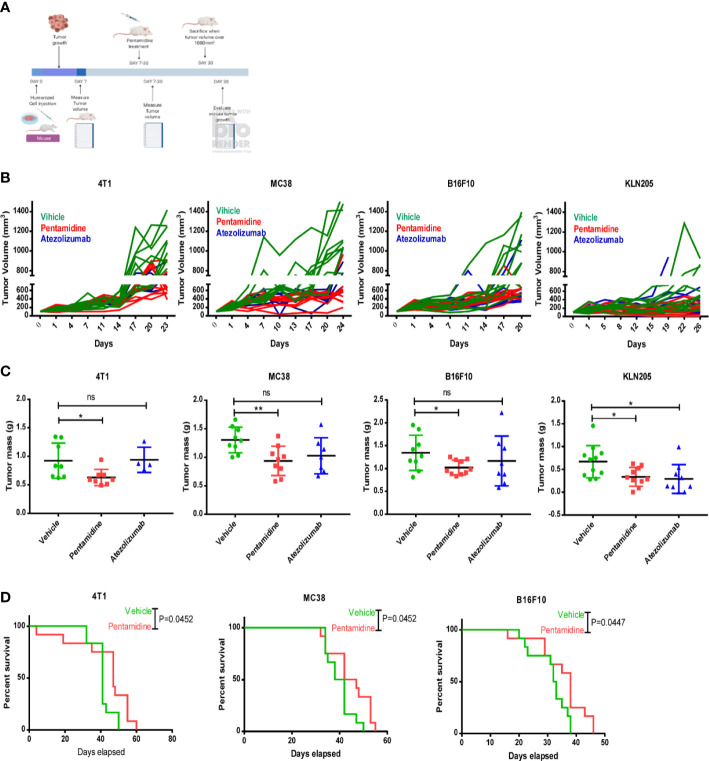
Pentamidine inhibits tumor growth in PD-L1 humanized murine cancer cell allograft models. **(A)** Schematic of the *in vivo* cell-derived allograft syngeneic model development and treatment with pentamidine (10 mg/kg, i.p.) or atezolizumab (5 mg/kg, i.p.). Quantification of tumor growth curves **(B)** and tumor mass **(C)** for 4T1, MC38, B16F10, and KLN205 cell-derived allograft tumors. Survival analysis of the pentamidine-treated group is shown (vehicle, n = 12 mice; pentamidine-treated mice, n = 12 mice) **(D)**. Results were pooled from two independent experiments, with p-values determined by a two-sided log-rank test. *P <0.05; **P <0.01; ns, not significant; one-way ANOVA with a *post-hoc* Bonferroni test.

To assess the anti-cancer effects of pentamidine in vivo, we initially evaluated its toxicity in C57 mice. Female mice were intraperitoneally (i.p.) injected with either vehicle or pentamidine at doses of 0.5 or 5 mg/kg every 2 days, while male mice were treated with doses of 1, 5, or 10 mg/kg every 2 days. Mouse body weight was measured every 2 days, and mice were sacrificed after 16 days. The body weight, liver, and spleen weights of the treated C57 mice were assessed for toxicity testing. The results demonstrated no significant changes in body weight, spleen weight, or liver weight in pentamidine-treated mice when compared to untreated controls (**Figure S4A**). We then proceeded to use the PD-L1 humanized syngeneic mouse model to test the anti-tumor effect of pentamidine. Allograft tumors were generated in mice via inoculation with human PDL-1-expressing murine cancer cells (4T1, MC38, KLN205, and B16F10). The PD-L1 humanized murine tumor cell allograft-bearing mice were subsequently treated with pentamidine or atezolizumab. The in vivo experimental design is illustrated in **Figure 5A**. After approximately 20 days of treatment, the mice were sacrificed, and tumor volumes and tumor masses were analyzed. An independent animal experiment was performed to quantify survival rates. The tumor growth curve showed that pentamidine and atezolizumab remarkably attenuated tumor growth compared to vehicle-treated mice (**Figure 5B**). The tumor weight was also significantly decreased in the pentamidine-treated group (**Figure 5C**). Additionally, the pentamidine-treated mice showed a prolonged survival time when compared with the vehicle-treated group (**Figure 5D**). The toxicity of pentamidine and atezolizumab in the PD-L1 humanized syngeneic model was determined by examining the changes in mouse body weight and alterations in spleen and liver mass. No obvious necrosis in the mouse spleen and liver was identified (data are not shown). Similarly, the body weight, spleen, and liver mass showed no significant changes, aside from the B16F10 group, in which the spleen mass was significantly lower in the pentamidine-treated group compared with the vehicle group (**Figure S4B**).

### 3.5 Pentamidine induced T-cell-mediated anti-tumor effects *in vivo*

**Figure 6:**
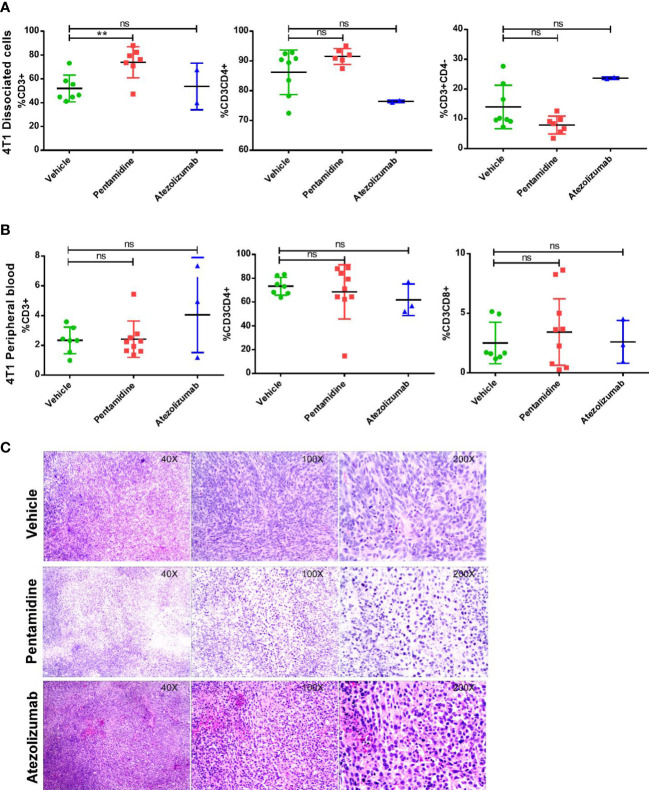
Analysis of immune components in the humanized allograft model. Populations of CD3+ lymphocytes, CD3+CD4+ T helper cells, and CD3+CD8+ cytotoxic T cells in peripheral blood or tumor microenvironment **(A, B)** in 4T1 cell-derived allograft tumors. **(C)** H&E staining shows tumor-infiltrating lymphocytes (TILs) in the humanized allograft tumor microenvironment. **p <0.01; ns, not significant; one-way ANOVA with a *post-hoc* Bonferroni test.

We hypothesized that pentamidine curbs the ability of the cancer cells to destroy T cells by binding with PD-L1 expressed by humanized murine tumor cells, thereby allowing T cells to infiltrate the tumor and initiate an anti-tumor immune response. To examine whether pentamidine initiates an anti-tumor immune response by activating T cells through blockade of tumor-derived PD-L1, we measured the population of helper T cells (TH) and cytotoxic T cells in peripheral blood and the tumor microenvironment by flow cytometry. In our study, we observed that in the tumor-dissociated single-cell pool (**Figure 6A**), only the CD3+ T cell population demonstrated a significant increase in the pentamidine-treated group. However, the other immune cell populations, such as CD3+CD4+ helper T cells and CD3+CD8+ cytotoxic T cells, exhibited no significant changes in either tumor-dissociated or peripheral samples (**Figure 6B**). H&E staining of tumor tissue sections was performed to investigate tumor-infiltrating lymphocytes (TILs) using a wide-field microscope (**Figure 6C**). Pentamidine treatment significantly increased TILs in PD-L1 humanized 4T1 syngeneic mouse allograft tumors (**Figure S5A**); however, no remarkable changes with respect to TILs were observed in PD-L1 humanized MC38 and B16F10 syngeneic murine allograft tumors (**Figures S5B, C**).

**Figure 7:**
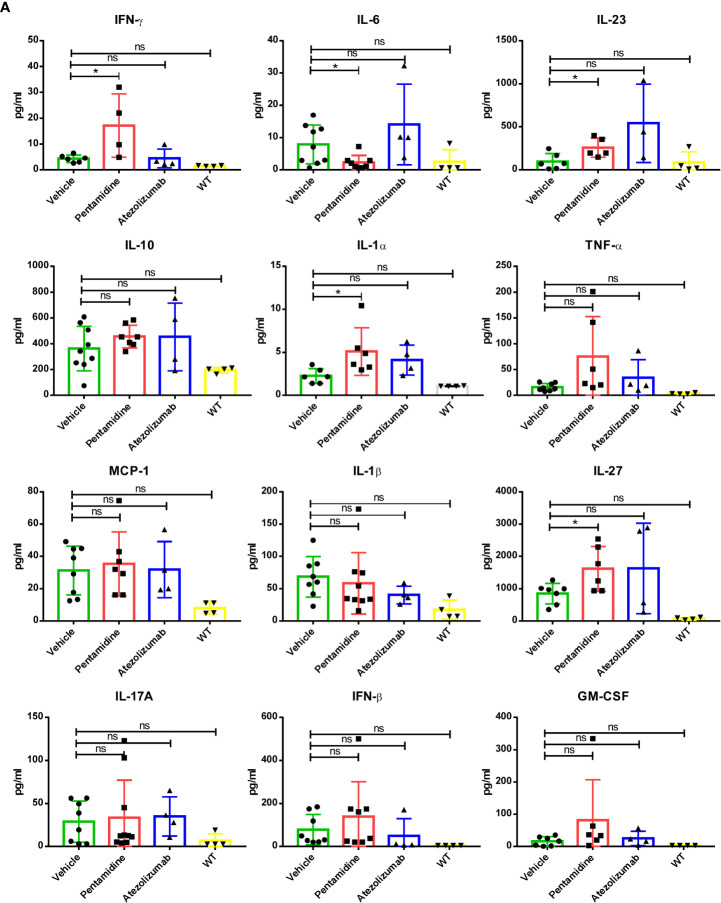
**(A)** Inflammation-related cytokine network in the humanized allograft model of 4T1 cell-derived allograft tumors. *p <0.05; ns, not significant; one-way ANOVA with a *post-hoc* Bonferroni test.

Characterizing the cytokine network provides an indirect way to monitor the immune response to cancer (23). Therefore, we conducted a multi-cytokine panel, including IL-1α, IL-1β, IL-6, IL-10, IL-12p70, IL-17A, IL-23, IL-27, MCP-1, IFN-β, IFN-γ, TNF-α, and GM-CSF to evaluate the level of inflammatory cytokines in the serum of syngeneic mice bearing PD-L1 humanized murine tumor allografts with or without pentamidine or atezolizumab treatment. Pentamidine treatment increased the levels of specific cytokines, such as IFN-γ, IL-23, IL-1α, and IL-27, while reducing the level of IL-6 in the serum of PD-L1 humanized 4T1 allograft-bearing mice as compared to the vehicle-treated group (**Figure 7A**). The production of IFN-γ can increase the host’s response to cancer cells by inducing CD8 T-cell differentiation and precursor proliferation (24). Although pentamidine did not increase the TILs in PD-L1 humanized MC38 and KLN205 syngeneic mouse allograft tumors, the level of IFN-γ in the serum of pentamidine-treated mice was higher than that in the serum of the vehicle-treated group (**Figures S6A, B**). However, pentamidine failed to significantly alter IFN-γ production in PD-L1 humanized B16F10 allograft tumor-bearing mice (**Figure S6C**).

**Figure 8:**
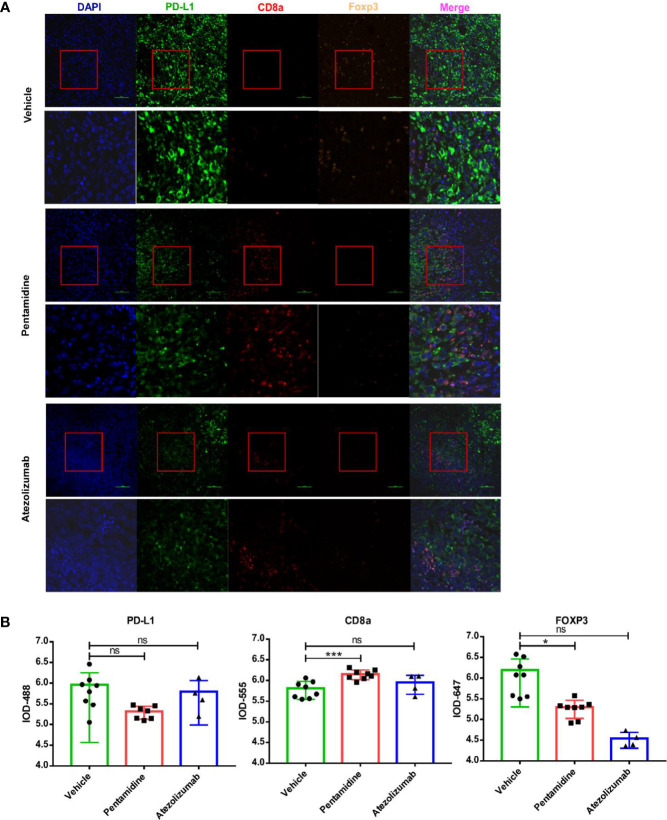
**(A)** Quantification of mouse CD8a, FOXP3, and human/mouse PD-L1 by fluorescent multiplex immunohistochemistry (mIHC). **(B)** Quantification of integrated optical density (IOD) of mouse CD8a, FOXP3, and human/mouse PD-L1. *p <0.05, ***p <0.001; ns, not significant; one-way ANOVA with a *post-hoc* Bonferroni test.

The expression of CD8, Foxp3, and PD-L1 in the tumor microenvironment is regarded as predictors for prognosis and response markers to immunotherapy in melanoma, breast cancer, NSCLC, and gastric cancer (25–27). As PD-L1 expression levels and the ratio of CD8+/FoxP3+ T cells can be used to characterize tumor immunity conditions (28), we performed a fluorescent multiplex immunohistochemistry (mIHC) assay to determine the expression of PD-L1 together with anti-tumor CD8 T cells and immune-suppressive FOXP3 positive Treg cells in PD-L1 humanized 4T1 syngeneic mouse allograft tumors (**Figure 8A**). With the aid of tyramide signal amplification and DAPI staining, the integrated optical density (IOD) of the fluorescent signals was evaluated using Image-Pro Plus 6.0 (Media Cybernetics, Silver Spring, USA) software (**Figure S7**). Our results showed that CD8 expression levels were elevated while FOXP3 expression levels were decreased after treatment with pentamidine compared with the vehicle group (**Figure 8B**).

## 4 Discussion

One of the mechanisms by which tumor cells avoid immune surveillance is the elevated expression of PD-L1, which inhibits T-cell activation by directly binding to PD-1. Thus, the PD-L1/PD-1 axis functions as a major contributor to T-cell exhaustion and has emerged as a potential target for cancer immunotherapy. In fact, the activation of the immune system to combat cancer has become a focus through the FDA approval of therapeutic monoclonal antibodies able to target critical immune checkpoint proteins, such as CTLA4, PD-1, and PD-L1 (29, 30). These monoclonal antibodies reinvigorate exhausted T cells, which can then infiltrate into the tumor microenvironment and initiate an anti-tumor response. However, only a subset of cancer patients respond to these immune-checkpoint inhibitors, indicating the need for extensive immune-oncology research to illuminate the underlying mechanisms governing tumor resistance to checkpoint inhibitors (29, 31). The mAbs that target the PD-1/PD-L1 interaction have certain limitations, including a long half-life, high cost, no oral bioavailability, and low diffusion and permeability (32, 33). Therefore, the development of small-molecule inhibitors for blocking the PD-1/PD-L1 pathway would be a welcome approach that could provide greater benefits to cancer immunotherapy. The resolution of the structural interaction between PD-1 and PD-L1 has made it possible to perform computer docking studies for virtual screening and predict molecules that may interrupt the interaction (13). Thus far, several small-molecule inhibitors of the PD-1/PD-L1 pathway have been investigated in clinical trials. These inhibitors include CA-170 (Curis Inc., NCT02812875), the macrocyclic peptide BMS-986189 (Bristol-Myers Squibb, NCT02739373), MAX-10181 (Maxinovel Pty., Ltd., NCT04122339), and INCB086550 (Incyte Corporation, NCT03762447). Until now, several reviews have summarized the development of small-molecule inhibitors of the PD-1/PD-L1 interaction as cancer immunotherapy (34–36). In the present study, we identified pentamidine, an existing antimicrobial agent, as a small-molecule inhibitor of PD-L1. Moreover, we found that pentamidine exerts anti-cancer effects by activating T-cell-mediated anti-tumor responses.

In search of small molecule inhibitors of the PD-1/PD-L1 axis, our first virtual screening of a compound library revealed a set of compounds showing binding affinity with PD-L1. Subsequent experimental findings indicated that pentamidine induced *in vitro* T-cell-mediated cancer cell cytotoxicity at a very low concentration (0.5 μM) but did not induce cytotoxic effects in normal cells even at a concentration of 10 μM. This led to the selection of pentamidine as a potential candidate to pursue further experiments. The sepharose-bead pull-down assay and SPR binding analysis showed that pentamidine selectively binds to human and mouse PD-L1, but not to PD-1. The docking model study further revealed the specific amino acids of PD-L1 that engage in interaction with pentamidine. The IC50 of pentamidine for blocking the interaction between PD-1/PD-L1 is 2.597 g/ml, which is comparable to the commercial small molecule BMS-1, which has an IC50 of 1.156 g/ml.

PD-1/PD-L1 signaling counteracts the CD28-TCR signaling pathway, leading to the suppression of T-cell proliferation. When PD-1 on the T cell surface interacts with PD-L1 on tumor cells, the phosphorylation of TCR signaling mediators such as Zap-70, Ras, and Akt decreases. This results in the inactivation of key transcription factors such as AP-1 or NFAT (18), ultimately leading to a reduction in T-cell proliferation, activation, effector functions, and survival. PD-1/PD-L1 blockade can reinvigorate exhausted T cells and restore their proliferation and cytotoxic function (19). To further elucidate the mechanisms underlying the anti-cancer effects of pentamidine, we generated a series of reporter cell lines, such as Jurkat T cells expressing NFAT-luc and NFAT-luc-PD-1, and 293T cells expressing TCR activator or TCR activator-PDL-1. The finding that the co-culture of Jurkat-NFAT-luc-PD-1 with 293T-TCR activator-PD-L1 cells led to reduced NFAT-luc signals indicated the inhibition of T-cell proliferation resulting from the PD-1–PD-L1 interaction. Treatment with pentamidine or atezolizumab restored the T-cell proliferation as revealed by increased NFAT-luc signals, suggesting that pentamidine disrupted the PD-1/PD-L1 signal axis and recovered activated T cells. The T-cell activating effects of pentamidine were comparable to those of the FDA approved PD-L1 antagonist atezolizumab. Like the results of the reporter cell lines, incubation with pentamidine or atezolizumab increased the cytotoxicity of various human-PD-L1 expressing cancer cell lines (H1975, H1937, A375, and HCT116) when co-cultured with human primary T cells expressing PD-1 endogenously. These findings suggest that disengaging PD-1 and PD-L1 by blocking PD-L1 with pentamidine facilitates T-cell-mediated tumor cell death.

T-cell effector cytokines can activate T cells to eliminate the target cancer cells. IFN-γ can increase the host’s response to tumors by decreasing tumor cell proliferation, arresting cell cycle, or inducing tumor cell apoptosis *via* ROS. IFN-γ also inhibits the function of certain suppressive immune cells, including tumor‐associated macrophages (TAMs) (37), myeloid‐derived suppressor cells (MDSCs) (38), and regulatory CD4+ T cells (Tregs) (39). On the other hand, IFN-γ plays a pivotal role during the immune evasion process by promoting angiogenesis, tumorigenesis, and immunosuppressive molecules (40, 41). IFN-γ mostly elicits PD‐L1 expression in cancer cells through the IFN-γ/JAK signaling pathway; indeed, IFN-γ treatment followed by PD‐L1 blockade was shown to result in enhanced anti-cancer T-cell cytotoxicity (42). In our co-culture system, we used cancer cells with relatively high PD-L1 expression levels. The increased IFN-γ production observed after T cells interacted with tumor cells not only provides evidence of T-cell activation but also demonstrates the anti-cancer effect of pentamidine as a PD-L1 blockade. The IFN-γ production serves as a more clinically relevant indicator in PD-L1 blockade studies (43). Cytokine production in the co-culture system indicated that both pentamidine and atezolizumab treatments showed an anti-cancer effect through the activation of T cells and the elimination of cancer cells. Besides IFN-γ, TNF-α, perforin, and Granzyme B production were elevated upon treatment of PD-L1-expressing tumor cells and primary T cells with pentamidine. In the inhibitory tumor microenvironment, T cells show an “exhausted” phenotype; production of IFN-γ and TNF-α was shown to maintain cytotoxic T cell stimulation and increase the cytotoxic effects of CD8+ T and NK cells (44). Production of perforin and Granzyme B is essential for effector CD8+ T-cell cytotoxic activity against cancer cells. The functional potential of the T-cell anti-cancer response was then assessed according to the production of IFN-γ, TNF-α, perforin, and Granzyme B. Our findings indicated that PD-L1 blockade by pentamidine enhanced the cytotoxic potential of T cells in all tested cancer cell lines.

The syngeneic murine allograft tumor model serves as a useful platform for evaluating the effects of immune checkpoint inhibitors. It has been demonstrated that murine PD-1 binds *in vitro* to both murine and human PD-L1, while human PD-1 binds to PD-L1 of each species (20). The crystal structure of the murine PD-1 interaction with the human PD-L1 complex (PDB:3BIK) further supports this notion. The cross-species binding affinities resemble those of same-species binding, suggesting the feasibility of such interactions. Despite the conservation of amino acid sequences between hPD-L1 and mPD-L1, their overall structures exhibit crucial differences that determine interactions with anti-PD-L1 agents (21).

Numerous studies have confirmed the possibility of interactions between murine PD-1 and human PD-L1. In a study, MC-38-Hpd-L1 cells were subcutaneously inoculated into C57BL/6 mice, and tumor-bearing mice were treated with an anti-hPD-L1 antibody *via* an intraperitoneal route (22). Furthermore, in this literature, a screening system for PD1/PD-L1 signaling in wild-type BALB/c mice was developed using murine PD-1 and human PD-L1 (45). Our SPR affinity study aligns with these findings, as pentamidine exhibits a higher affinity compared to murine PD-L1. Given the evidence from the literature and our own research, we chose to employ an hPD-L1 syngeneic allograft model for our study.

We conducted a PD-L1 humanized syngeneic murine tumor allograft study to verify the antagonist effect of pentamidine against human PD-L1 *in vivo.* The results indicated that pentamidine decreased the volume of allograft tumors and extended the survival of tumor-bearing mice without exhibiting liver or spleen toxicity. Moreover, further investigation showed that pentamidine boosted the immune response to tumor growth by increasing the population of CD8a+ T cells and decreasing Treg abundance, thus prolonging the survival of tumor-bearing mice. Populations of CD3+ T cells and the ratio of CD4+/CD8+ T cells in the peripheral or tumor microenvironment were quantified to assess the immune response to allograft tumors. In the 4T1 syngeneic murine model, CD3+ T-cell abundance in the peripheral blood increased after pentamidine treatment, indicating that pentamidine enhanced T-cell proliferation and persistence. Histochemical analysis of allograft tumors showed that pentamidine-treated allograft tumors were infiltrated and surrounded by lymphocytes only in 4T1-derived tumors but not in tumors generated from other cell lines. It would be better to analyze the TIL in other tumor allograft models early, as the allograft tumor cells might already have been eliminated by the immune response at the time of analysis. Taken together, our findings suggest that pentamidine, an FDA-approved compound used for over 25 years (46, 47), can be repurposed to target PD-L1 as an immuno-oncology therapeutic.

While our study has provided valuable insights into the potential of pentamidine as a repurposed drug for PD-1/PD-L1 blockade in both “cold” and “hot” allograft tumor models, there are several limitations that should be acknowledged. One such limitation is the use of allo-activation of T cells in our *in vitro* experiments. Allo-activation of T cells may not be directly relevant to the clinical setting, as it does not involve antigen-specific T-cell activation.

Future studies could benefit from establishing organoids derived from cancer patients and extracting infiltrating T cells from the tumor microenvironment or peripheral blood. Or we should employ TCR-transduced T-cell co-culture with antigen-expressing tumor cells, such as TCR (1G4) and NY-ESO-1, which may offer a more comprehensive understanding of pentamidine’s role in modulating T-cell activation and function. By evaluating the therapeutic effect of pentamidine on PD-L1 checkpoint inhibition using those more clinically relevant models, we may better understand the potential of pentamidine as a repurposed drug for PD-1/PD-L1 blockade in the context of antigen-specific T-cell activation. By acknowledging these limitations, we provide a more balanced perspective on our study’s findings and highlight areas for future research to build upon our preliminary results.

## 5 Conclusion

We have discovered pentamidine as a small-molecule antagonist of PD-L1. Pentamidine directly binds to the PD-L1 protein, but not PD1, and increases T-cell-mediated cytotoxicity to cancer cells. Treatment with pentamidine reduced tumor mass and volume in PD-L1 humanized syngeneic mouse allograft tumor models. Histochemical analysis of allograft tumors revealed increased T-cell infiltration in tumor tissues. Pentamidine, as a novel PD-L1 antagonist, may overcome the limits of monoclonal antibody therapies and efficiently increase checkpoint blockade in cancer patients.

## Data availability statement

The raw data supporting the conclusions of this article will be made available by the authors, without undue reservation.

## Ethics statement

The animal study was reviewed and approved by China-US (Henan) Hormel Cancer Institute Ethics Committee.

## Author contributions

TG, XT, M-HL, and ZD were involved in study concept and design, acquisition of data, analysis, and interpretation of data, and drafting of the manuscript. TG, XT, YW, WY, and WL performed experiments. MS, RZ, MW, QG, TL, CZ, and KL supported the data analysis and materials. TG, XT, M-HL, JK, and ZD wrote the manuscript. ZD and M-HL supervised the study. The assignment order of the co-first authors was determined by the contribution rate. All authors contributed to the article and approved the submitted version.

## Supplementary material

Supplementary Figure 1 PD-L1 expression levels in various cancer cell lines **(A)** and pentamidine toxicity on primary T cells (right panel) and H1975 lung cancer cells (left panel) **(B)**. Effects of PD-1/PD-L1 inhibitors screened from a docking model-predicted compound library **(C)**. **P < 0.01; ns, not significant; one-way ANOVA with *post hoc* Bonferroni test.

Supplementary Figure 2 Establishment of cellular reporter system for PD-1/PD-L1 checkpoint inhibition. Flow cytometry detection of PD-1 expression after lentivirus infection **(A)** and single cell clone seeding **(B)**. NFAT-dependent luciferase activity of Jurkat-NFAT-PD-1 cell clones evaluated following PMA (50 ng/mL) stimulation **(C)**. Flow cytometry detection of PD-L1 expression in 293T-TCR-activator PD-L1 cell clones **(D)**; red color: WT-293T cell; blue color, 293T-TCR-activator PD-L1 cell labeled by IgG-PE; yellow color, 293T-TCR-activator PD-L1 cell labeled by anti-PD-L1-PE.

Supplementary Figure 3 Generation of PD-L1 humanized murine cancer cell lines. Schematic illustrating the creation of murine PD-L1 knock-out and human PD-L1 expression **(A)**. Flow cytometry detection of murine PD-L1 knock-out in MC38 cells using anti-murine CD274-APC to detect murine PD-L1, controlled by rat IgG-APC isotype **(C)**. Humanization of PD-L1 in 4T1, B16F10, MC38, and KLN205 murine cells. Anti-murine CD274-APC and anti-human CD274-PE used to detect murine and human PD-L1; controlled by rat IgG-APC, mouse IgG-PE isotype **(C)**.

Supplementary Figure 4
*In vivo* pentamidine toxicity in mice. Body weight, liver weight, and spleen weight calculated for C57 male (right panel) and female mice (left panel) **(A)**. Body weight, liver weight, and spleen weight in 4T1, MC38, B16F10, and KLN205 syngeneic cell-derived allograft tumor experiments **(B)**.

Supplementary Figure 5 H&E staining for identifying tumor-infiltrating lymphocytes (TILs) in 4T1 allograft tumor tissue **(A)**, MC38 allograft tumor tissue **(B)**, and B16F10 allograft tumor tissue **(C)**. *P < 0.05; **P < 0.01; ns, not significant; one-way ANOVA with *post hoc* Bonferroni test.

Supplementary Figure 6 Cytokine network analysis with serum derived from humanized murine cancer cell allograft mice: MC38 **(A)**, KLN205 **(B)**, and B16F10 **(C)**. *P < 0.05; ns, not significant; one-way ANOVA with *post hoc* Bonferroni test.

Supplementary Figure 7 CD8a, FOXP3, and PD-L1 expression levels in 4T1 cell-derived tumor tissues of humanized cancer cell allograft mice assessed in the vehicle-treated group **(A)**, pentamidine-treated group **(B)**, and the atezolizumab-treated group **(C)**.
