# Supplementary figures and images for: Repurposing pentamidine for cancer immunotherapy by targeting the PD1/PD-L1 immune checkpoint
Source: Front Immunol. 2023 May 2;14:1145028. doi: 10.3389/fimmu.2023.1145028 (PMC10185823; doi:10.3389/fimmu.2023.1145028)

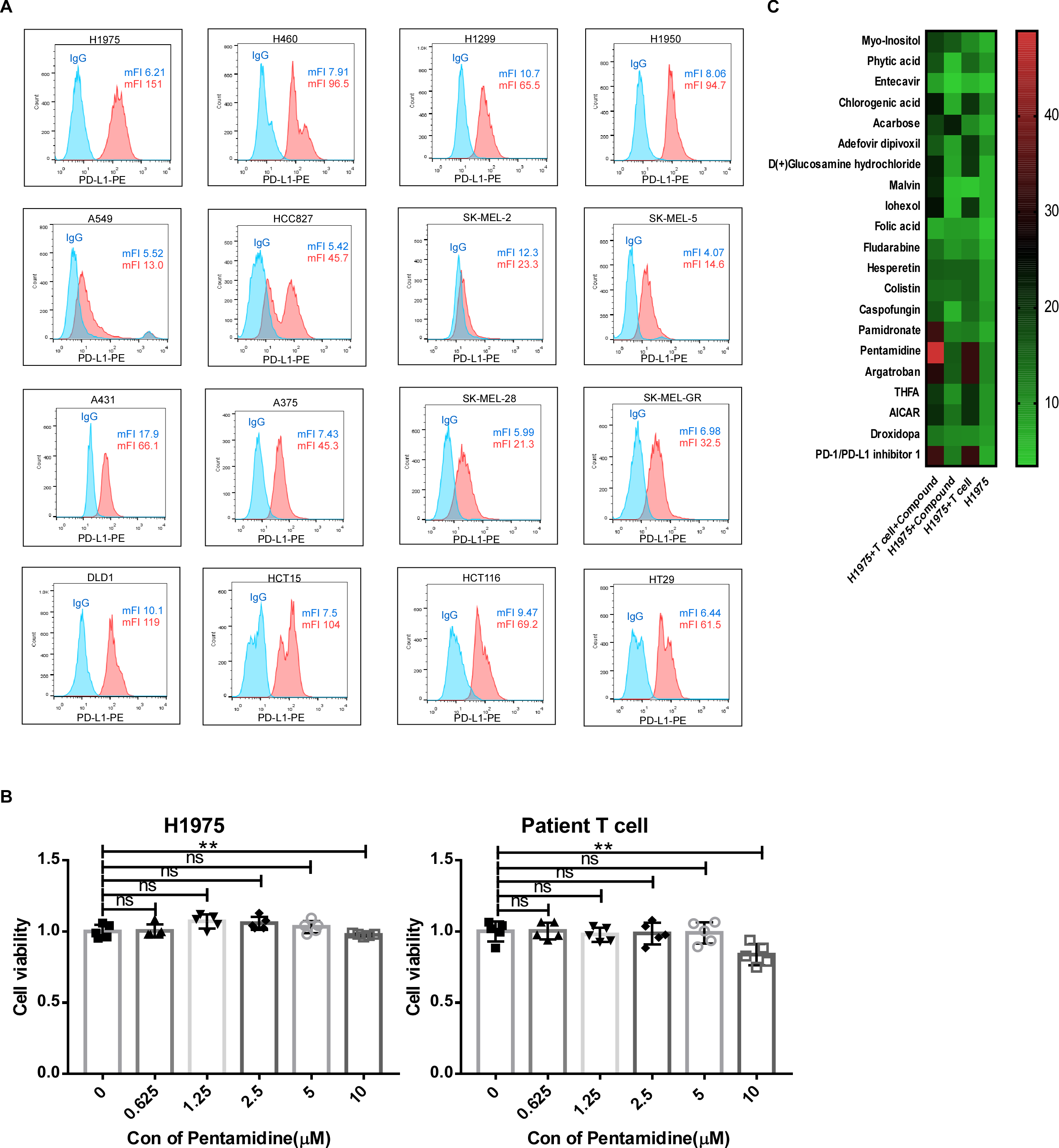

Supplement: Supplementary Figure 1 — PD-L1 expression levels in various cancer cell lines (A) and pentamidine toxicity on primary T cells (right panel) and H1975 lung cancer cells (left panel) (B). Effects of PD-1/PD-L1 inhibitors screened from a docking model-predicted compound library (C). **P < 0.01; ns, not significant; one-way ANOVA with post hoc Bonferroni test. [file Image_1.tif]

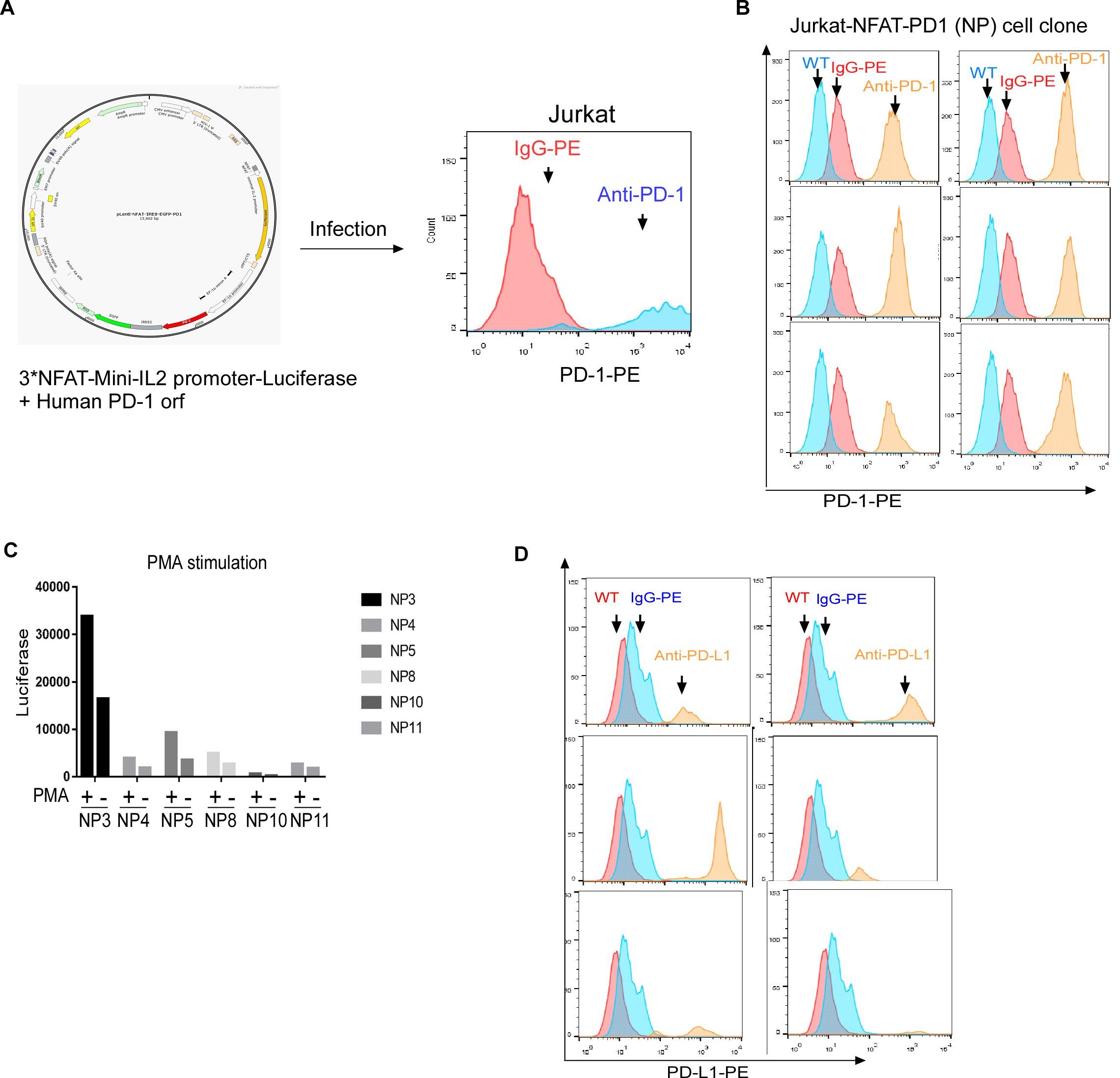

Supplement: Supplementary Figure 2 — Establishment of cellular reporter system for PD-1/PD-L1 checkpoint inhibition. Flow cytometry detection of PD-1 expression after lentivirus infection (A) and single cell clone seeding (B). NFAT-dependent luciferase activity of Jurkat-NFAT-PD-1 cell clones evaluated following PMA (50 ng/mL) stimulation (C). Flow cytometry detection of PD-L1 expression in 293T-TCR-activator PD-L1 cell clones (D); red color: WT-293T cell; blue color, 293T-TCR-activator PD-L1 cell labeled by IgG-PE; yellow color, 293T-TCR-activator PD-L1 cell labeled by anti-PD-L1-PE. [file Image_2.tif]

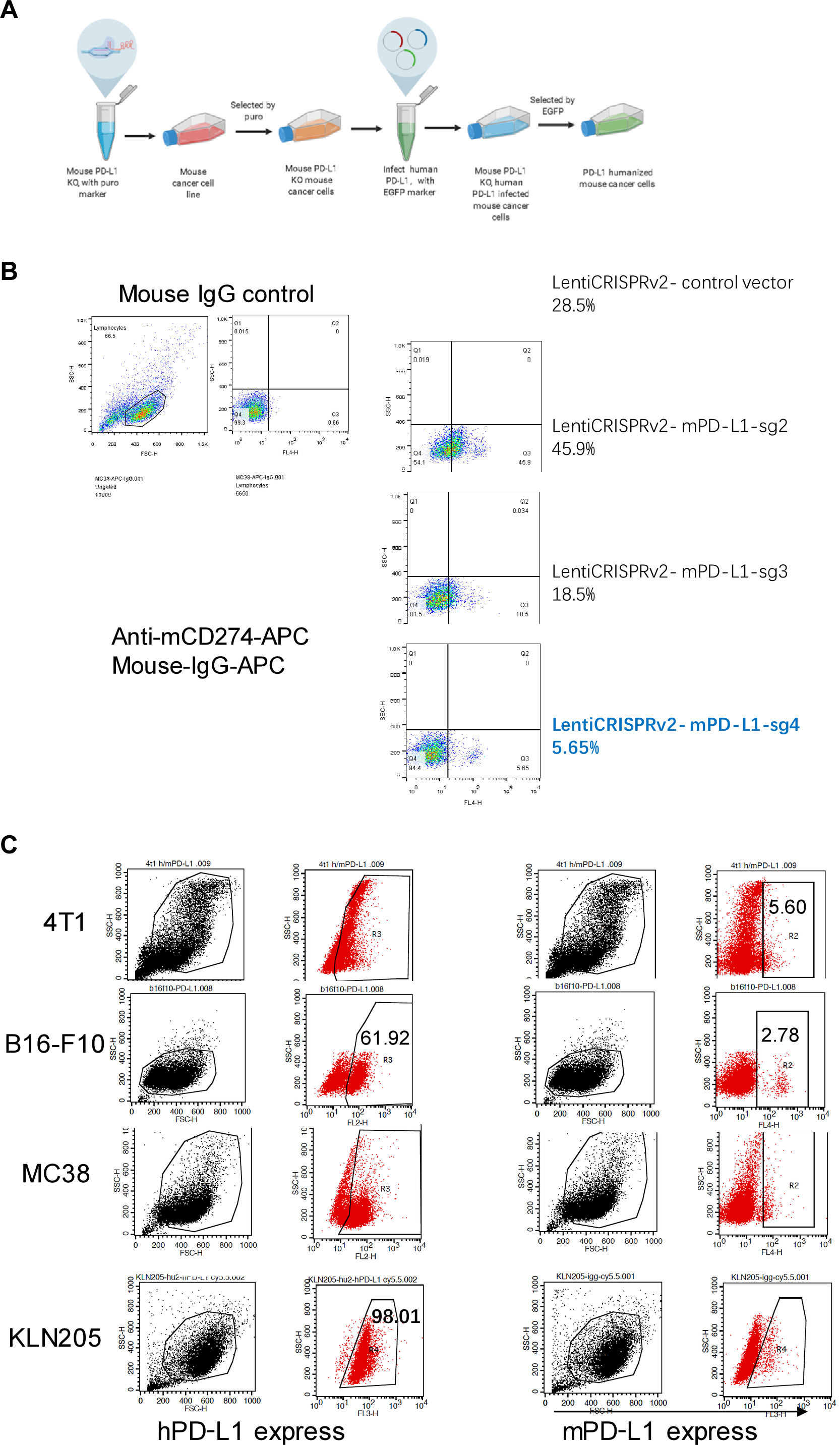

Supplement: Supplementary Figure 3 — Generation of PD-L1 humanized murine cancer cell lines. Schematic illustrating the creation of murine PD-L1 knock-out and human PD-L1 expression (A). Flow cytometry detection of murine PD-L1 knock-out in MC38 cells using anti-murine CD274-APC to detect murine PD-L1, controlled by rat IgG-APC isotype (C). Humanization of PD-L1 in 4T1, B16F10, MC38, and KLN205 murine cells. Anti-murine CD274-APC and anti-human CD274-PE used to detect murine and human PD-L1; controlled by rat IgG-APC, mouse IgG-PE isotype (C). [file Image_3.tif]

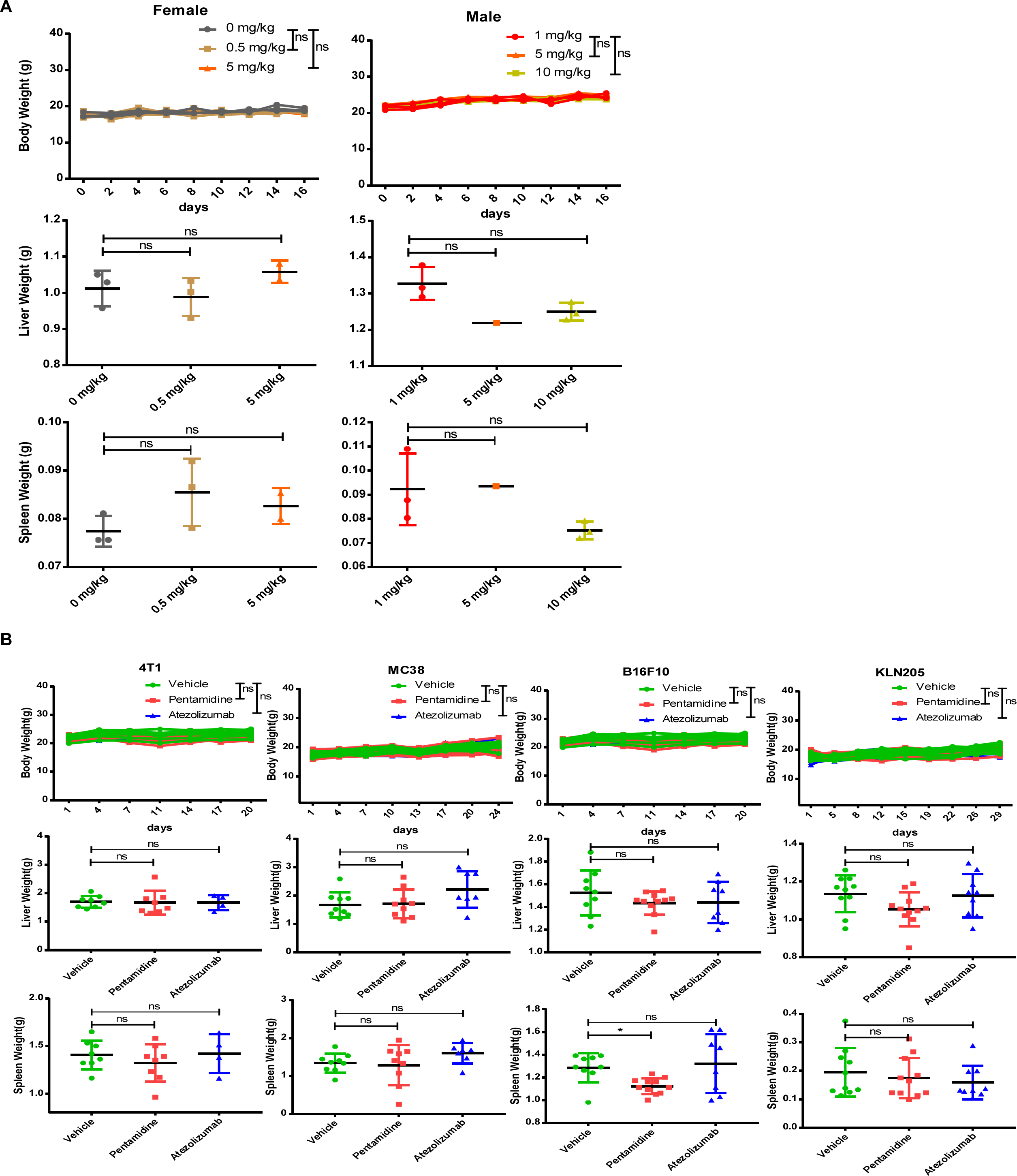

Supplement: Supplementary Figure 4 — In vivo pentamidine toxicity in mice. Body weight, liver weight, and spleen weight calculated for C57 male (right panel) and female mice (left panel) (A). Body weight, liver weight, and spleen weight in 4T1, MC38, B16F10, and KLN205 syngeneic cell-derived allograft tumor experiments (B). [file Image_4.tif]

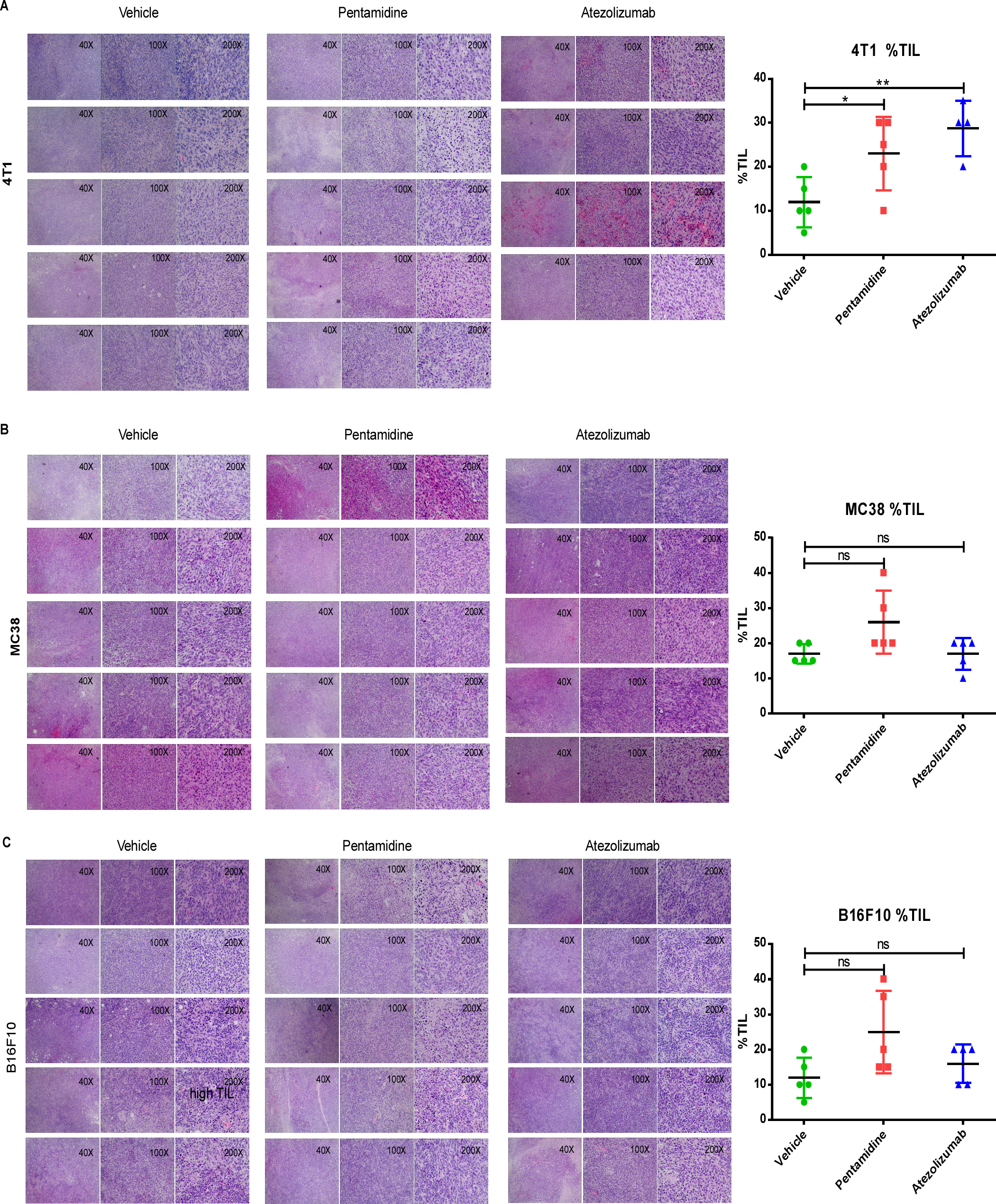

Supplement: Supplementary Figure 5 — H&E staining for identifying tumor-infiltrating lymphocytes (TILs) in 4T1 allograft tumor tissue (A), MC38 allograft tumor tissue (B), and B16F10 allograft tumor tissue (C). *P < 0.05; **P < 0.01; ns, not significant; one-way ANOVA with post hoc Bonferroni test. [file Image_5.tif]

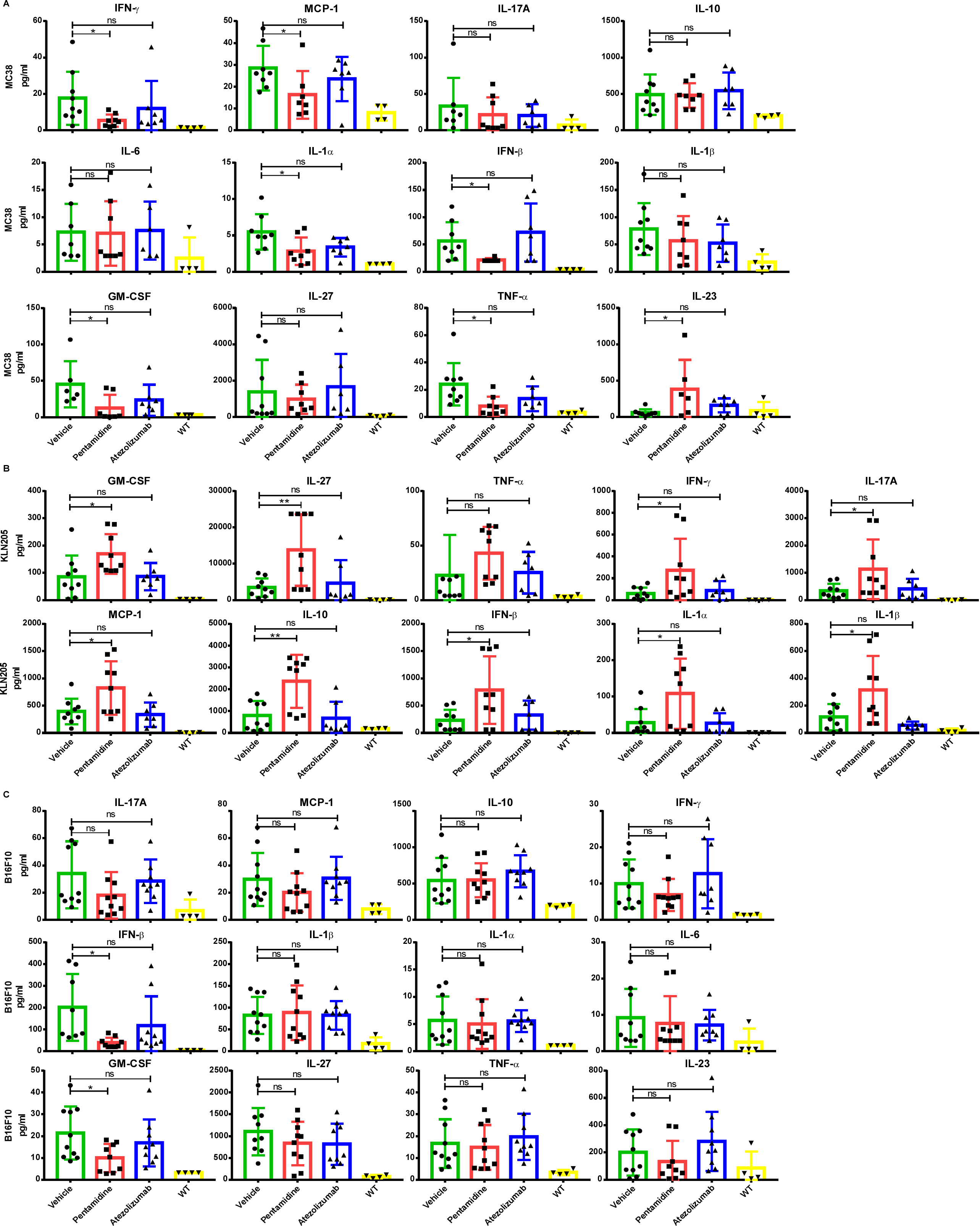

Supplement: Supplementary Figure 6 — Cytokine network analysis with serum derived from humanized murine cancer cell allograft mice: MC38 (A), KLN205 (B), and B16F10 (C). *P < 0.05; ns, not significant; one-way ANOVA with post hoc Bonferroni test. [file Image_6.tif]

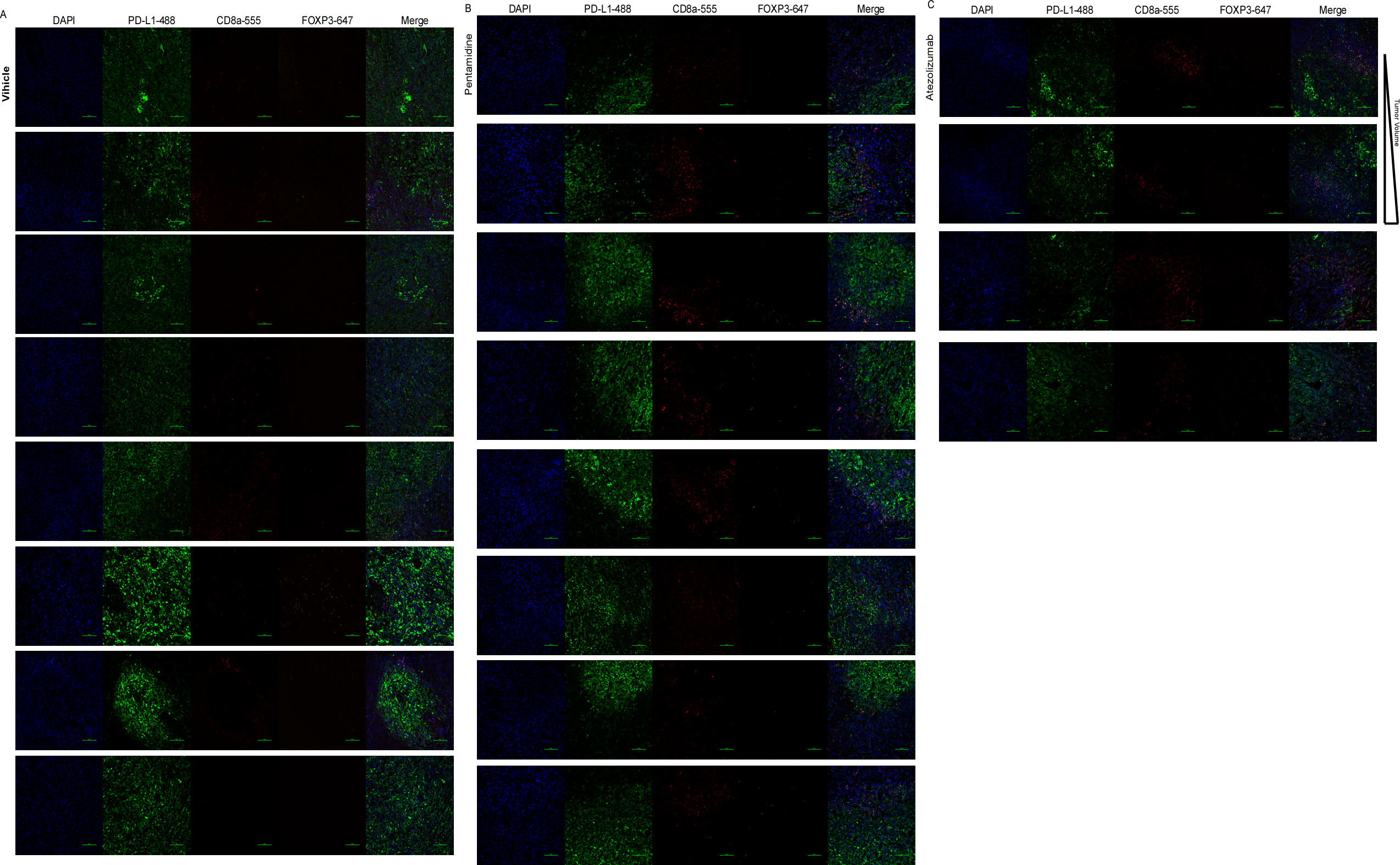

Supplement: Supplementary Figure 7 — CD8a, FOXP3, and PD-L1 expression levels in 4T1 cell-derived tumor tissues of humanized cancer cell allograft mice assessed in the vehicle-treated group (A), pentamidine-treated group (B), and the atezolizumab-treated group (C). [file Image_7.tif]
